# A new perspective on atrial tachycardia-induced cardiomyopathy: The misdiagnosis of epigastric pain in an 11-year-old girl

**DOI:** 10.1016/j.radcr.2024.09.086

**Published:** 2024-09-27

**Authors:** Min Zhang, Yong Zhang, Xiaoxiao Cao

**Affiliations:** The Children's Heart Center, Wuhan Children's Hospital, Tongji Medical College, Huazhong University of Science & Technology, 100 Hongkong Road, Jiangan District, Wuhan, Hubei, China

**Keywords:** Epigastric pain, Atrial tachycardia-induced cardiomyopathy, Radiofrequency catheter ablation, Children

## Abstract

Epigastric pain can be a common clinical manifestation of many diseases, but severe subxiphoid pain caused by tachycardia-induced cardiomyopathy is extremely rare in children. Therefore, the clinical manifestations of the disease are diverse, and improving early detection and treatment of the disease can avoid possible risks. In the case, we report an 11-year-old girl who was initially diagnosed with acute gastritis in a local hospital, but was later diagnosed with atrial tachycardia-induced cardiomyopathy in our hospital after active diagnosis and effective treatment, suggesting early detection and intervention is possible to prevent subsequent serious events.

## Introduction

Focal atrial tachycardia accounts for approximately 10% of supraventricular tachycardia. It has been reported that atrial tachycardia and other supraventricular tachycardias with ventricular tachycardia can cause cardiomyopathy. However, in children with atrial tachycardia cardiomyopathy, left ventricular function can be significantly improved by effective control of ventricular rate. Therefore, early diagnosis and active treatment of children with atrial tachycardia-induced cardiomyopathy are very important. Atrial-induced cardiomyopathy in children is rare, and its clinical manifestations are varied, including epigastric pain as a rarer clinical manifestation. Therefore, it is necessary to strengthen the understanding, accurate diagnosis and active treatment of atrial tachycardia-induced cardiomyopathy in children with special clinical manifestations to prevent critical events. In the paper, we summarize the case of an 11-year-old girl with atrial tachycardia-induced cardiomyopathy with epigastric pain as a clinical manifestation, who gradually recovered after correct diagnosis and effective treatment.

## Case presentation

**Fig. 1:**
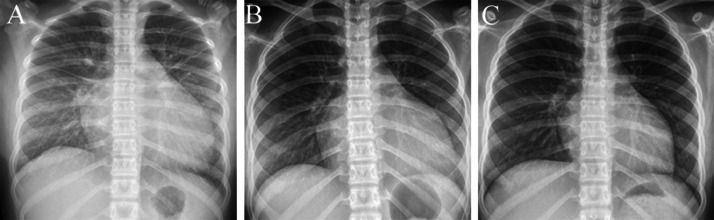
Interstitial pulmonary edema in both lungs with enlargement of the entire heart, with enlargement of the left heart dominating (A). The interstitial pulmonary edema has disappeared, but the entire heart remains enlarged (B). The lungs are normal and the cardiac shadow is significantly improved (C).

**Fig. 2:**
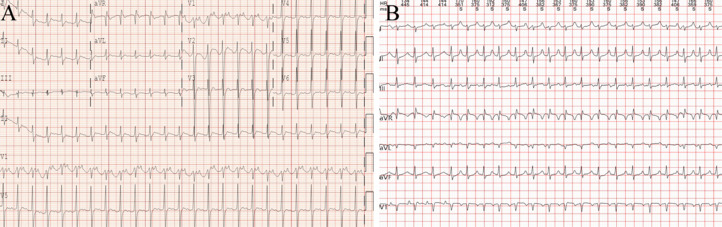
A 12-lead electrocardiogram shows the atrial rhythm (A). The 24-hour Holter electrocardiogram shows atrial tachycardia (B).

An 11-year-old girl presented with persistent and intense subxiphoid pain for 5 days, which was excruciating, painful, and interfered with sleep, accompanied by nausea and occasional vomiting of watery gastric contents, occasional dizziness, and without cough or fever. Acute gastritis was diagnosed in a local hospital, but gastroduodenoscopy showed no inflammatory changes in the stomach and duodenum. The child was then transferred to our hospital, where we subjected her to a physical examination: conscious, painful face, no obvious dry and wet rales in the lungs according to auscultation; There was no bulge in the precordial area, the heart rate was 131 beats/minute, irregular heartbeat, deep and dull heart sound, slightly swollen abdomen, obvious tenderness under the xiphoid process, no rebound pain, and normal limb movement. The child underwent a detailed ancillary examination. The results of the laboratory test are as follows: Creatinine 67.8 umol/L (range 27-66 umol/L), Uric acid 679 umol/L (range 208-428 umol/L), Total bilirubin 29.5 umol/L (range 0-21 umol/L), Direct bilirubin 14.9 umol/L (range 0-6.8 umol/L), Alanine transaminase 83 U/L (range 7-30 U/L), Aspartate aminotransferase 92 U/L (range 14-44 U/L), Prothrombin time 16.3 seconds (range 10.2-13.4 seconds), Prothrombin time - international standard ratio 1.51 (range 0.88-1.16), DD-dimer 6.51 mg/L (range 0-0.55 mg/L FEU). Chest X-ray showed pleural effusion (Fig. 1), abdominal CT effusion, Echocardiography revealed an LVEF of 15%, indicating very low cardiac function. The 12-lead electrocardiogram (ECG) showed normal sinus rhythm, but the Holter electrocardiogram showed that atrial rhythm predominates and atrial tachycardia can be detected (Fig. 2). The patient was treated with dobutamine and deslanoside to increase myocardial contractility and amiodarone to correct heart rate. The patient's ejection fraction gradually recovered to 46%, but the ECG monitor still showed uninterrupted atrial rhythm with chest discomfort. After 2 weeks of oral administration of the drug, the left ejection fraction of the heart again decreased to 29%. Therefore, we considered that antiarrhythmic drugs were not effective and performed radiofrequency ablation under general anesthesia with parental consent.

**Fig. 3:**
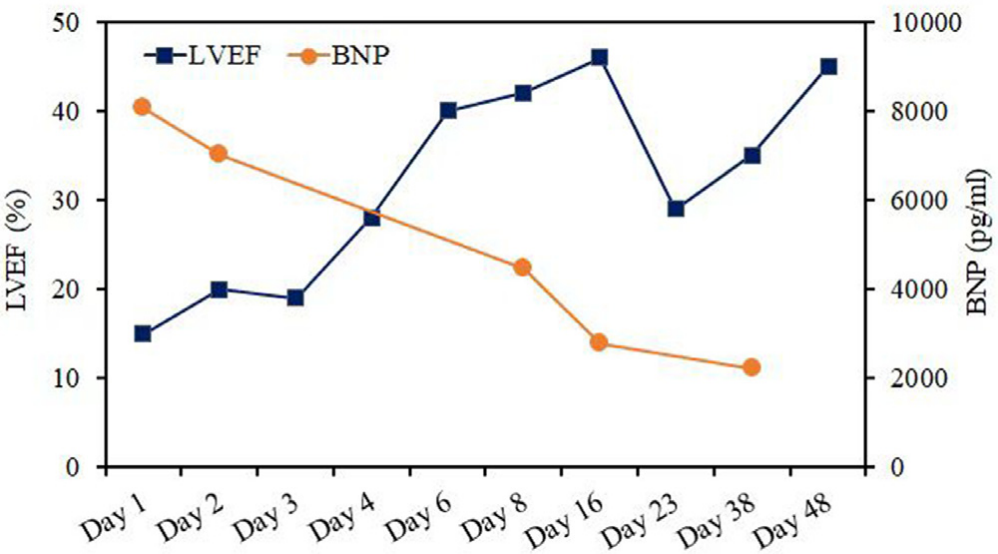
During treatment, LVEF gradually improved and BNP gradually normalized.

During radiofrequency catheter ablation, the ectopic pacemaker was located on the right pulmonary vein, and radiofrequency energy was delivered with a target temperature of 43°C, a power of 30 W, and a flow rate of 17 mL/minute. At this point, the heart rhythm changed to sinus rhythm, so the catheter ablation was repeated a total of 4 times within 60 seconds. After isoproterenol testing and atrial stimulation, there was no atrial rhythm, indicating that the child's operation was successfully completed. After 2 months of follow-up after radiofrequency catheter ablation, the patient was asymptomatic in daily life and maintained sinus rhythm, and the LVEF increased to 43% (Fig. 3).

## Discussion

Atrial tachycardia is the most common cause of supraventricular tachycardia in children, with a lower likelihood of spontaneous resolution in children over 3 years of age [1]. Conventional antiarrhythmic drugs are ineffective in treating atrial tachycardia, and prolonged recurrent episodes can lead to significant ventricular systolic dysfunction, a major cause of arrhythmic cardiomyopathy in children [[2], [3], [4]]. In this case, the child had shown physical deterioration in the past, but she did not go to the hospital for a detailed examination, and only when pain in the upper abdomen caused by this heart failure appeared, the specific disease was identified as Atrial tachycardia-induced cardiomyopathy. During hospitalization, the child's cardiac function gradually improved after treatment with deslanoside and amiodarone, but she still experienced intermittent chest tightness, and the ECG monitor showed an atrial rhythm at that time. The child's symptoms resolved and sinus rhythm was maintained until radiofrequency catheter ablation of atrial tachycardia was performed.

Atrial tachycardia-induced cardiomyopathy results in a range of clinical symptoms, with epigastric pain being a rare primary manifestation in children. Therefore, a quick and accurate diagnosis of upper abdominal pain in children is crucial for pediatricians to accurately diagnose the condition [5]. The child's atrial rhythm was not detected on the 12-lead ECG but was identified by the Holter electrocardiogram. Therefore, it is crucial to improve the interpretation of ECG and perform additional examinations such as Holter electrocardiogram and echocardiography for a more comprehensive diagnosis. The cause of this child's multi-organ damage was insufficient blood flow to various organs caused by heart failure due to cardiomyopathy. Therefore, with the gradual improvement of cardiac function, the functions of various organs also gradually improved and returned to normal. Therefore, in children with multiple organ dysfunction, it is necessary to look at the cause from multiple angles, paying particular attention to whether the cause is related to heart disease.

Atrial tachycardia-induced cardiomyopathy is a reversible cardiomyopathy that can be treated with antiarrhythmic drugs or radiofrequency catheter ablation to restore sinus rhythm, followed by a gradual return to normal cardiac function. Therefore, early detection of atrial tachycardia with special clinical manifestations and early treatment are helpful to prevent atrial tachycardia-induced cardiomyopathy [6,7]. At present, the child has only received radiofrequency catheter ablation for 2 months, and it is necessary to dynamically observe whether the child's atrial tachycardia recurs and whether the systolic cardiac function changes, which requires follow-up care.

## Patient consent

Written informed consent was obtained from the patient for publication of this case report and accompanying images. A copy of the written consent is available for review by the Editor-in-Chief of this journal on request.
